# Predictive factors for the efficacy of brolucizumab in refractory polypoidal choroidal vasculopathy following aflibercept resistance

**DOI:** 10.1371/journal.pone.0326018

**Published:** 2025-11-04

**Authors:** Akie Yoshinaga, Kohdai Kitamoto, Shuichiro Aoki, Ryo Terao, Tatsuya Inoue, Ryo Obata, Keiko Azuma

**Affiliations:** 1 Department of Ophthalmology, Graduate School of Medicine and Faculty of Medicine, The University of Tokyo, 7-3-1 Hongo, Bunkyo-ku, Tokyo, Japan; 2 Department of Ophthalmology and Showa General Hospital, Hanakoganei, Kodaira, Tokyo, Japan; 3 Department of Ophthalmology and Micro-Technology, Yokohama City University School of Medicine, 4-57 Urafune-cho, Minami-ku, Yokohama, Kanagawa, Japan; 4 Department of Ophthalmology and Toranomon Hospital, 2-2-2 Toranomon, Minato-ku, Tokyo, Japan; Akita University: Akita Daigaku, JAPAN

## Abstract

**Purpose:**

To identify predictors of extension of the injection interval beyond 8 weeks at the 24-month visit after switching to brolucizumab in aflibercept-resistant polypoidal choroidal vasculopathy (PCV).

**Design:**

Retrospective observational study

**Methods:**

17 eyes of 16 patients with persistent or recurrent exudation on aflibercept were switched to intravitreal brolucizumab and managed with a treat-and-extend (T&E) regimen with a minimum 8-week interval after loading. The primary outcome contrasted extension (>8 weeks) versus non-extension (≤8 weeks) at month 24. Prespecified predictors were early central choroidal thickness (CCT) change from baseline to the switch visit (A0 to A1; ≥ 40% reduction) and pachychoroid. Associations were tested with Fisher’s exact tests and Firth-penalized logistic regression with the event defined as extension.

**Results:**

At 24 months, 6 of 17 eyes (35%) achieved extension. A ≥ 40% early CCT reduction occurred in 0 of 6 extension eyes versus 7 of 11 non-extension eyes (Fisher exact two-sided P ≈ 0.035). In the Firth model (event = extension), < 40% CCT reduction strongly predicted extension (odds ratio 38.5; profile-likelihood 95% CI 2.0–10,000; LR P = 0.004). Non-pachychoroid showed the same direction with wide CIs (odds ratio 14.3; 95% CI 0.99–2,174; LR P = 0.006). Model fit was significant (LR χ² = 15.19, P = 0.0005) and discrimination was good (apparent AUC ≈ 0.97). We prespecified a parsimonious two-predictor model to limit overfitting; adding age, sex, prior photodynamic therapy, or number of prior aflibercept injections did not materially change coefficients or improve AICc (ΔAICc < 2).

**Conclusions:**

Eyes without marked early choroidal thinning (<40% CCT reduction at A1) were more likely to extend, whereas marked thinning (≥40%) signaled difficulty extending under T&E regimen after switching to brolucizumab. Given the small sample and few events, estimates should be interpreted cautiously and considered hypothesis-generating, and warrant prospective external validation studies.

## Introduction

Neovascular age-related macular degeneration (nAMD) is a leading cause of blindness in industrialized countries and a major public health concern among the aging population [1]. It is characterized by macular neovascularization, leading to the accumulation of subretinal and intraretinal fluid, which contributes to progressive central vision loss [2]. Optical coherence tomography (OCT) allows for the quantification of disease activity by assessing central macular thickness (CMT), central choroidal thickness (CCT) and fluid changes within the retinal layers [3]. The introduction of intravitreal anti-vascular endothelial growth factor (VEGF) therapy has revolutionized the management of nAMD, significantly improving visual outcomes and reducing the risk of blindness [4]. Despite the success of anti-VEGF therapy, treatment response in nAMD is highly heterogeneous. While some patients achieve long-term disease control with a limited number of injections, others require frequent monthly treatments, yet still fail to achieve adequate disease suppression [5,6]. One of the major limitations of anti-VEGF therapy is tachyphylaxis, a phenomenon in which repeated intravitreal injections lead to a gradual reduction in drug efficacy over time [7,8]. Studies have shown that up to 8.9% of nAMD patients develop tachyphylaxis to intravitreal aflibercept (Eylea; Regeneron Pharmaceuticals, Tarrytown, NY, USA; Bayer AG, Leverkusen, Germany) with an annual incidence of approximately 3% [9]. In clinical practice, several strategies have been explored to overcome treatment resistance and extend injection intervals, including switching between anti-VEGF agents, high-dose therapy, combination treatments, and optimizing treatment regimens, though the long-term efficacy of these approaches remains uncertain [10–13].

Unlike typical nAMD, polypoidal choroidal vasculopathy (PCV) often shows reduced durability to anti-VEGF therapy, with persistent or recurrent fluid despite aflibercept and a need for short maintenance intervals or adjunct photodynamic therapy (PDT) in a subset of cases [7,9]. These features likely reflect pachychoroid-related choroidal hyperpermeability and dilated Haller vessels [14–16].

Brolucizumab (Beovu, Novartis Pharma AG, Basel, Switzerland) is a single-chain antibody fragment (scFv) with a molecular weight of 26 kDa, characterized by high VEGF-A affinity and superior tissue penetration [17–19]. The pivotal HAWK and HARRIER trials demonstrated that brolucizumab provides comparable visual gains to aflibercept while achieving superior fluid resolution [17,18]. Additionally, brolucizumab showed a higher probability of extending the treatment interval to 12 weeks, thereby reducing treatment burden [18]. However, the predictive factors associated with successful extension of the injection interval beyond 8 weeks following a switch from intravitreal aflibercept (IVA) to brolucizumab remain poorly understood. Given the heterogeneity in treatment response, identifying these factors is essential for optimizing personalized treatment strategies and minimizing the burden of frequent injections. While brolucizumab achieved these efficacy outcomes, a small but notable risk of intraocular inflammation (IOI)—including retinal vasculitis and/or retinal vascular occlusion—has been reported in phase 3 trials and post-marketing studies, warranting careful patient selection and vigilant monitoring [17,18].

In this study, extension was a priori defined as maintaining an injection interval >8 weeks at 24 months (A2) under a treat-and-extend (T&E) regimen after switching to intravitreal brolucizumab. Therefore, we aimed to identify predictors of extension (>8 weeks at A2) under a T&E regimen after switching to intravitreal brolucizumab (IVBr) in aflibercept-resistant PCV.

## Materials and methods

### Study population

This retrospective study was conducted in accordance with the tenets of the Declaration of Helsinki and was approved by the Institutional Review Board of the University of Tokyo, Japan (Ethics Committee ID number: 2217). The requirement for written informed consent was waived due to the retrospective nature of the study; an opt-out process was implemented, and patients who declined the use of their medical records for research purposes were excluded. All data were de-identified prior to analysis.

We reviewed consecutive medical records of patients who visited the University of Tokyo Hospital between March 2021 and January 2023. Data used in this retrospective study were accessed from March 2021 to January 2023. All patients underwent comprehensive ophthalmologic examinations, including best-corrected visual acuity (BCVA), intraocular pressure (IOP), slit-lamp biomicroscopy, fundus examination, and spectral-domain optical coherence tomography (SD-OCT; Spectralis, Heidelberg Engineering, Germany).

The cohort comprised eyes with PCV that were anti-VEGF–naïve at baseline and began treatment with IVA. After a standard three-injection loading phase and, in most cases, additional IVA maintenance (with some eyes also receiving PDT), eyes were switched to IVBr for persistent or recurrent macular fluid under IVA. All eyes were then managed with a T&E regimen (post-loading minimum 8-week interval) and completed 24 months of follow-up.

### Inclusion and exclusion criteria

Inclusion criteria were: (1) a diagnosis of PCV based on the criteria defined by the Asia-Pacific Ocular Imaging Society (APOIS) PCV Workgroup [14] (i.e., protruding orange-red lesions on fundus examination or polypoidal lesions on indocyanine green angiography [ICGA]); (2) persistent or recurrent exudative findings after ≥3 consecutive IVA injections; (3) switch to IVBr due to insufficient fluid resolution, visual acuity deterioration, subretinal hemorrhage, or inability to extend treatment intervals; and (4) completion of two years of follow-up after switching to IVBr.

Exclusion criteria included the presence of other macular pathologies such as inflammatory or hereditary retinal disease, chorioretinal atrophy, previous laser treatment, significant media opacities interfering with imaging, glaucoma, or other retinal or optic nerve disorders.

The safety population comprised 18 eyes from 17 patients (including one bilateral patient) that received ≥1 IVBr and was used for adverse-event reporting and exposure counts (total injections). The efficacy population comprised 17 eyes from 16 patients (including one bilateral patient) after excluding one unilateral eye that developed intraocular inflammation after the first IVBr; this set was used for outcome analyses.

### Diagnosis of pachychoroid

All patients were evaluated for pachychoroid features using multimodal imaging (reduced fundus tessellation on color photography, dilated outer choroidal vessels on enhanced depth imaging OCT (EDI-OCT), and regional choroidal vascular hyperpermeability [CVH] on ICGA). For classification, we used a prespecified multimodal rule requiring ≥2 of 3 features: (1) CCT ≥ 200 µm at baseline (A0); (2) dilated Haller’s layer vessels (pachyvessels) on EDI-OCT; and (3) CVH on ICGA. [15,20,21].

CCT was measured at the foveal center on EDI-OCT from the outer border of the RPE/Bruch’s complex to the choroid–scleral interface. Given the lack of a universally accepted CCT cut-off and known variability with age/axial length/ethnicity, 200 µm was used as a pragmatic anchor within a multimodal definition, in line with recommendations to avoid thickness-only definitions [22].

### Intravitreal Injection

All patients received intravitreal injections of aflibercept (2.0 mg/0.05 mL) and brolucizumab (6.0 mg/0.05 mL) [23,24]. After administering a topical anesthetic (0.4% oxybuprocaine hydrochloride; Benoxil™, Santen Pharmaceutical Co., Tokyo, Japan), the injections were performed using the standard pars plana approach (3.5 mm posterior to the limbus) with a 30-gauge needle under sterile conditions in a procedure room.

### Treatment Protocol and Group Classification

Following a three-dose loading phase, dosing intervals were governed by a fixed minimum of 8 weeks; intervals <8 weeks were not used. Thereafter, intervals were adjusted in 2-week steps within 8–12 weeks based on OCT and BCVA at each visit: Extend (8 → 10 → 12) required a dry macula (no IRF/SRF), no new hemorrhage, and stable BCVA (<0.1 logarithmic minimum angle of resolution (logMAR)). Maintain was permitted only for trace, stable sub-RPE fluid without IRF/SRF and with stable BCVA. Shorten meant reverting any extended interval back to 8 weeks in the presence of IRF/SRF recurrence, new hemorrhage, or a BCVA decline ≥0.1 logMAR attributable to exudation. Extension in the presence of IRF/SRF was not permitted. No re-switching to other anti-VEGF agents or adjunctive PDT occurred during follow-up.

The main outcome was the ability to extend the treatment interval beyond 8 weeks at the 2-year mark. Patients were categorized into two groups:

Non-extenders (≤8 weeks): Patients who showed fluid recurrence within 8 weeks, preventing interval extension.

Extenders (>8 weeks): Patients who maintained a dry macula for more than 8 weeks and achieved treatment extension.

### Outcomes and imaging evaluation

Primary outcome. Extension status at A2 categorized as >8 weeks (extension) vs ≤ 8 weeks (non-extension) under a T&E regimen.

Evaluation was conducted at three timepoints:

A0: Prior to first IVA injection

A1: Prior to first IVBr injection

A2: Two years after switching to IVBr

Secondary outcomes. BCVA (converted to logMAR for analysis), presence of SRF, IRF, or sub-RPE fluid, and CCT. CCT was measured using EDI-OCT as the distance from Bruch’s membrane to the inner scleral surface. Measurements were performed by two independent macula specialists, and the mean values were used for analysis.

In addition to continuous analyses, BCVA (logMAR) changes were categorized using clinically meaningful thresholds (≥0.1, ≥ 0.2, and ≥0.3 logMAR improvement) and compared between the extension (>8 weeks at A2) and non-extension (≤8 weeks) groups using two-sided Fisher’s exact tests. We considered ≥0.1 logMAR as a minimal clinically important difference.

### Treatment exposure/ Injection counts

IVBr injection counts over 24 months were summarized overall and by extension group as median [interquartile range] and mean ± SD. Counts refer to IVBr after the switch; no re-switching or adjunct PDT occurred. The analysis unit was the eye. The safety population included all eyes receiving ≥1 IVBr; the efficacy population excluded the IOI eye.

### Statistical analyses

Continuous variables were summarized as mean ± SD or median [IQR] when non-normal. Normality was assessed with the Shapiro–Wilk test (with Q–Q plot inspection) and homogeneity of variances with Levene’s test. Between-group differences were compared using Student’s t test when assumptions were met or the Mann–Whitney U test otherwise; within-eye/time comparisons used paired t tests or Wilcoxon signed-rank tests. Categorical variables were analyzed with Pearson’s χ² test or Fisher’s exact test when any expected cell count was < 5.

For the primary analysis, we used Firth-penalized logistic regression to model extension >8 weeks at 24 months (A2) with a prespecified two-predictor model: early CCT change (≥40% reduction from A0 → A1) and pachychoroid. Because of the small sample and potential (quasi-)separation, we used Firth-penalized logistic regression to reduce small-sample bias and obtain finite, stable odds ratios. We report profile-likelihood 95% CIs and LR-test P-values.

Model fit was summarized by the likelihood-ratio χ² and Pearson/Deviance goodness-of-fit tests; discrimination was assessed with the area under the ROC curve (AUC) computed from model-predicted probabilities.

To assess potential confounding, we added age, sex, PDT history, and pre-switch IVA number one at a time to the prespecified two-predictor Firth model (extension coded as 1). A variable was deemed a confounder if it changed the CCT coefficient (log-odds) by ≥10% or reduced AICc by ≥2; none met these thresholds, so the two-predictor model was retained. All tests were two-sided with α = 0.05. Analyses were conducted in JMP Pro 17 (SAS Institute, Cary, NC, USA).

Given the retrospective, exploratory nature and small sample, no a priori power calculation was performed; instead, effect sizes with 95% CIs are presented.

## Results

Seventeen eyes from 16 patients (13 men, 3 women) were included in the efficacy analyses. Baseline characteristics are summarized in Table 1. The mean age was 73.0 ± 8.84 and 76.8 ± 7.14 years in the non-extension and extension groups, respectively (P = 0.38). (Table 1)

**Table 1:** Baseline characteristics by extension status at 24 months (A2). Values are mean ± SD unless otherwise indicated. Continuous variables were compared using t-tests when assumptions were met or Mann–Whitney U otherwise; categorical variables by χ² or Fisher’s exact (expected count <5). BCVA, best-corrected visual acuity (logMAR); CCT, central choroidal thickness (μm); PDT, photodynamic therapy; IVA, intravitreal aflibercept.

	Non-extension (≤8 weeks)	Extension (>8 weeks)	P value
Number of eyes	11	6	
Age (years)	73.0 ± 8.84	76.8 ± 7.14	0.38
Male/female	9/2	5/1	0.94
BCVA at A0 (logMAR)	0.14 ± 0.25	0.12 ± 0.20	0.85
BCVA at A1 (logMAR)	0.29 ± 0.31	0.24 ± 0.15	0.71
BCVA at A2 (logMAR)	0.39 ± 0.31	0.15 ± 0.22	0.11
CCT at A0 (μm)	195.0 ± 43.8	143.2 ± 39.7	0.030
CCT at A1 (μm)	113.0 ± 34.6	127.0 ± 49.0	0.50
Early CCT change A0 → A1 (%)	42 ± 11	13 ± 14	<.0001
≥40% CCT reduction A0 → A1, n/N (%)	7/11(64)	0/6(0)	0.0033
History of receiving PDT	6	1	0.36
Number of prior IVA doses before switching	30.0 ± 18.91	24.5 ± 28.93	0.64
Presence of pachychoroid	9	1	0.035

BCVA: Best-Corrected Visual Acuity, CCT: central choroidal thickness, PDT: photodynamic therapy, IVA: intravitreal aflibercept, *P < 0.05, compared between the groups

We analyzed 6 extension (>8 weeks) and 11 non-extension (≤8 weeks) eyes.

Mean BCVA (logMAR) did not differ between the extension (>8 weeks) and non-extension (≤8 weeks) groups at A0, A1, or A2; detailed values are provided in **Table 1**. An improvement of ≥0.1 logMAR was observed in 4/6 (67%) eyes in the extension group versus 1/11 (9%) in the non-extension group (Fisher’s exact P = 0.029). Improvements of ≥0.2 logMAR (1/6 vs 1/11) and ≥0.3 logMAR (0/6 vs 1/11) did not differ significantly (two-sided Fisher’s exact, P > 0.3 for both). These threshold-based findings were consistent with the direction of the continuous BCVA analyses.

**Fig 1:**
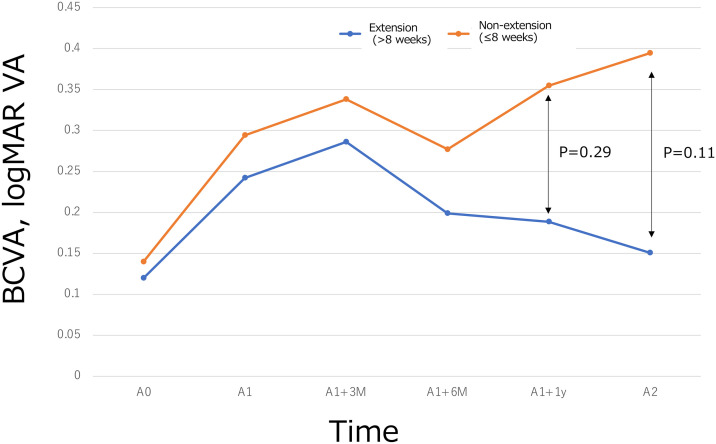
The changes in best-corrected visual acuity (BCVA, logMAR) at three key time points: before aflibercept (IVA) treatment (A0), before brolucizumab (IVBr) treatment (A1), and two years after switching to IVBr (A2). The data are presented for two groups: Group 1 (non-extension (≤8 weeks), requiring more frequent injections) and extension (>8 weeks), with extended treatment intervals). No significant differences in BCVA were observed between the groups at any of the given time points (P > 0.05 for all comparisons).

**Fig 1** shows a change in BCVA (logMAR). The rate of dry macula was 0% at baseline (A0 point), 0% at A1, and 35% at A2.

**Fig 2:**
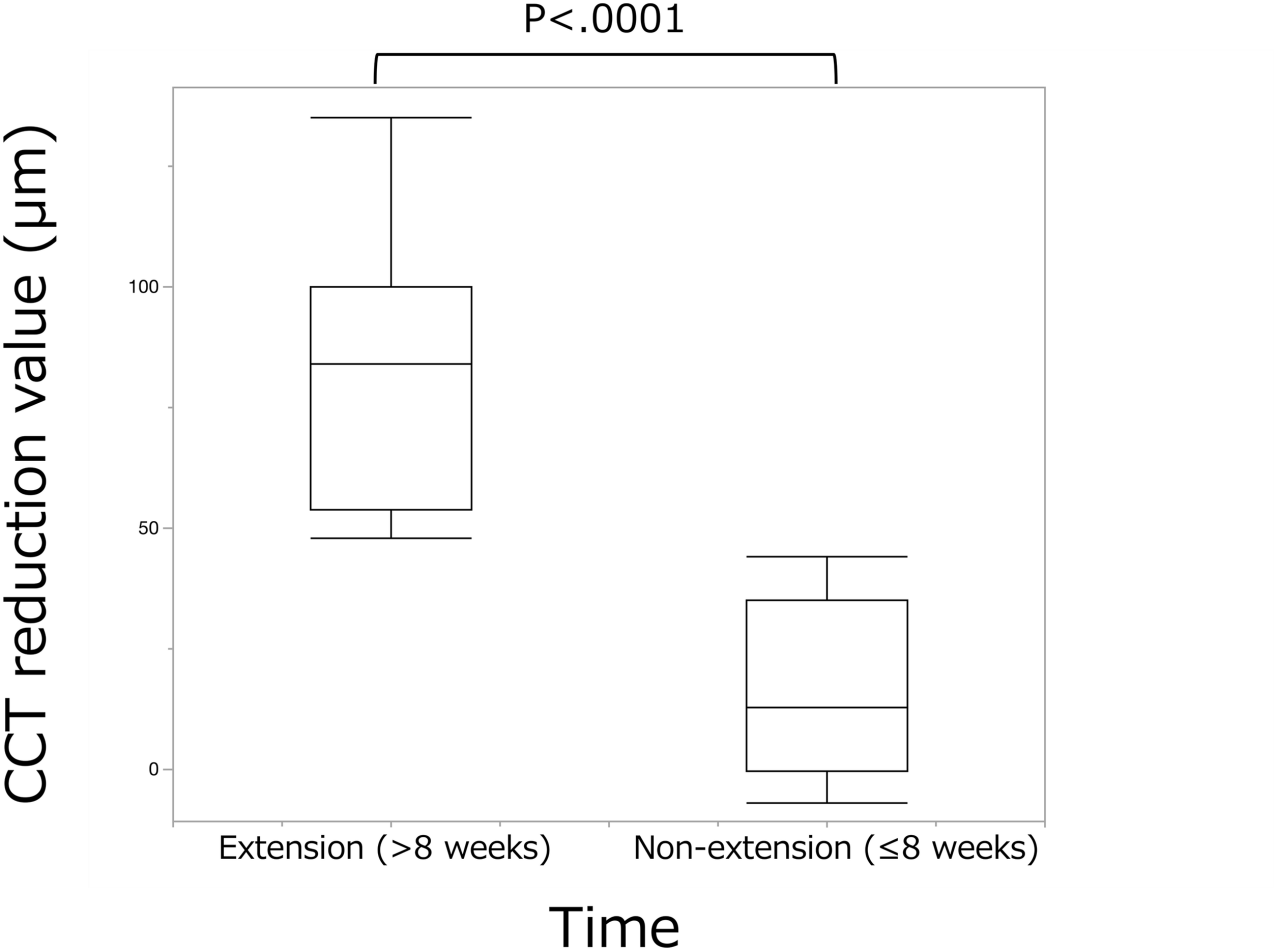
The changes in central choroidal thickness (CCT) at two key time points: before aflibercept (IVA) treatment (A0) and before brolucizumab (IVBr) treatment (A1). Group 1 (non-extension (≤8 weeks)) showed a significantly greater reduction in CCT from A0 to A1 compared to Group 2 (extension (>8 weeks)) (P < 0.0001, Mann–Whitney U test). A significant difference in CCT was observed between the two groups at A0 (P = 0.030, Mann–Whitney U test), but no significant difference was found at A1 (P = 0.50).

**Fig 3:**
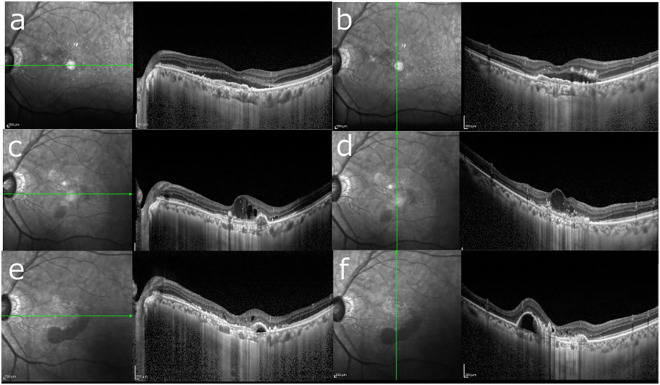
Representative case from the non-extension group (≤8 weeks). A 74-year-old man. (a, b) Baseline horizontal and vertical OCT scans before initiating aflibercept; CCT 195 µm. Despite 43 IVA injections, exudative changes persisted, indicating resistance. (c, d) Horizontal and vertical OCT scans immediately before switching to brolucizumab (A1); CCT 85 µm with residual activity. (e, f) At 24 months (A2) under a T&E regimen with brolucizumab, recurrent disease with associated hemorrhage is seen; CCT 89 µm. The injection interval could not be extended beyond 8 weeks for maintenance.

**Fig 4:**
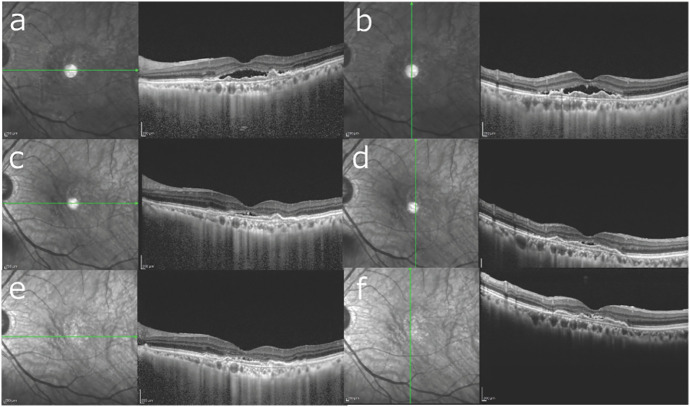
Representative case from the extension group (>8 weeks). An 85-year-old man. **(a, b)** Baseline horizontal and vertical OCT scans before initiating aflibercept; CCT 127 µm. Despite four consecutive IVA injections, exudative changes persisted. **(c, d)** Horizontal and vertical OCT scans immediately before switching to brolucizumab (A1); CCT 119 µm with residual activity. **(e, f)** At 24 months (A2) under a T&E regimen with brolucizumab, the macula is dry and the maintenance interval was > 8 weeks; CCT 122 µm.

At baseline (A0), mean CCT was greater in the non-extension (≤8 weeks) group than in the extension (>8 weeks) group (195.0 ± 43.8 µm vs 143.2 ± 39.7 µm; P = 0.030). At the switch visit (A1), CCT did not differ between groups (113.0 ± 34.6 µm vs 127.0 ± 49.0 µm; P = 0.50). The early CCT change from A0 → A1 was significantly larger (i.e., greater thinning) in the non-extension group (P < 0.0001; Fig 2). Figs 3–4 show representative cases from each group.

Across all eyes, the absolute CCT change from A0 to A1 had a median of 54 µm [IQR 25–85 µm; range −7–135 µm]. Three eyes (18%) showed decreases >100 µm; these heavily pre-treated eyes (pre-switch IVA 41, 43, 62 injections; one with prior PDT) all belonged to the non-extension group.

Over 24 months, the cohort received a median of 13 IVBr injections [IQR 12–14] overall (mean 12.71 ± 1.21). Eyes that maintained >8-week intervals (n = 6) received 12 injections [10–12] (mean 11.50 ± 1.23), whereas ≤8-week eyes (n = 11) received 13 [13–14] (mean 13.36 ± 0.50). Year-by-year medians were 7 [6–7] vs 7 [7–8] in year 1 and 5 [4–5] vs 6 [6–6] (extension vs non-extension). For safety reporting, total IVBr exposures were 217 injections (including one in the IOI eye); injections in the 17 analyzed eyes numbered 216.

Multivariable logistic regression was performed to identify factors associated with extension (>8 weeks) at the 2-year visit (Table 2).

**Table 2:** Predictors of extension (>8 weeks at 24 months) after switching to brolucizumab: Firth-penalized logistic regression.

Explanatory Variables	Extension (>8 weeks) at 24 months (A2)		
	OR	95%CI	P-value
CCT reduction <40% (A0 → A1)	38.5	2.0–10,000	0.004
Non-pachychoroid	14.3	0.99–2,174	0.006

Event = extension (>8 weeks) at the 24-month visit (A2). The prespecified model included two predictors: early CCT reduction ≥40% from A0 to A1 and pachychoroid. Odds ratios (OR) are shown with profile-likelihood 95% confidence intervals; P values from likelihood-ratio tests.Abbreviations: CCT, subfoveal choroidal thickness; A0, baseline; A1, switch visit; A2, 24-month visit; OR, odds ratio.

*P < 0.05, compared between the groups.

The prespecified two-predictor model used Firth-penalized logistic regression with event = extension, including early CCT change (<40% vs ≥ 40% reduction from A0 → A1) and pachychoroid. CCT reduction <40% strongly predicted extension (odds ratio 38.5; profile-likelihood 95% CI 2.0–10,000; LR P = 0.004). Non-pachychoroid showed the same direction with borderline precision (odds ratio 14.3; 95% CI 0.99–2,174; LR P = 0.006). Model fit was significant (LR χ² = 15.19, P = 0.0005), Pearson/Deviance goodness-of-fit tests were non-significant, and discrimination was good (apparent AUC ≈ 0.97). In sensitivity analyses adding age, sex, prior PDT, or number of pre-switch aflibercept injections one at a time, the CCT coefficient changed by <10% and AICc did not improve (ΔAICc < 2); therefore, the two-predictor specification was retained.

### Safety

Eighteen eyes from 17 patients with aflibercept-resistant PCV were switched to IVBr. One unilateral eye developed IOI 2 weeks after the first IVBr and was excluded from efficacy analyses; the IOI resolved within 1 week with topical corticosteroids, with no angiographic evidence of retinal vasculitis or retinal vascular occlusion. The efficacy cohort therefore comprised 17 eyes from 16 patients (including one bilateral patient). Over 24 months, 216 IVBr injections were administered in the 17 analyzed eyes (median 13 per eye); the excluded IOI eye received one injection, for a total of 217 IVBr exposures. Among the 17 analyzed eyes over 24 months, no further IOI occurred and no cases of retinal vasculitis, retinal vascular occlusion, endophthalmitis, or arterial thromboembolic events were observed.

## Discussion

In this retrospective cohort of aflibercept-resistant PCV switched to brolucizumab under a T&E regimen, we examined factors associated with extension of the injection interval (>8 weeks at 24 months). In Firth-penalized models with extension as the outcome, a ≥ 40% early reduction in CCT from baseline to the switch visit (A0 → A1) was associated with lower odds of extension—that is, eyes with marked early choroidal thinning were more likely to require maintenance at ≤8 weeks. Baseline pachychoroid showed a similar direction but with wide confidence intervals, indicating limited precision in this small sample. Taken together, on-treatment choroidal dynamics (early CCT change) appeared more informative than a static pachychoroid label when planning T&E intervals after switching to brolucizumab. Mechanistically, early CCT thinning likely captures an on-treatment choroidal state (decongestion/remodeling with borderline choriocapillaris/RPE reserve), whereas pachychoroid reflects a chronic trait; the former was more informative for interval planning after the IVBr switch.

Visual outcomes were broadly comparable between groups at A0–A2; however, a ≥ 0.1-logMAR improvement (a minimal clinically important difference) occurred more often in eyes that maintained >8-week intervals; these analyses were underpowered for larger thresholds and should be interpreted cautiously.

Several studies have investigated predictors of IVBr response in nAMD, including PCV [25–27]. Among them, Hirayama et al. [26] reported that fewer prior anti-VEGF injections and pre-switch CCT < 250 µm were associated with favorable outcomes at one year. In our cohort, all eyes already had pre-switch CCT < 250 µm (mean 118 ± 39 µm), precluding stratification by that absolute threshold. Because CCT typically decreases during anti-VEGF therapy [28], not all suboptimal responders have thick choroid. We therefore focused on change (A0 → A1) and found that greater early thinning was linked to lower odds of extension at 24 months. This is consistent with reports that greater baseline CCT—reflecting the pachychoroid milieu with dilated Haller vessels and choroidal hyperpermeability—correlates with shorter anti-VEGF durability in PCV, likely because anti-VEGF does not correct underlying choroidal congestion/hyperpermeability [16]. Mechanistically, CCT reduction after anti-VEGF may reflect decreased choroidal permeability, vascular regression, and remodeling [28,29], which could exacerbate ischemia or structural compromise; VEGF-dependency may therefore persist and durability remain limited despite potent suppression [29–31]. Thus, marked early CCT thinning probably denotes decongestion/remodeling with borderline CC/RPE reserve, so fluid tends to recur as the drug effect wanes, limiting interval extension. Baseline CCT remains relevant, but in our data on-treatment change provided greater predictive value than pre-switch thickness alone.

Baseline pachychoroid features showed a similar (imprecise) association with lower odds of extension, supporting the concept that pachychoroid morphology—thickening, CVH, and dilated outer choroidal vessels—can sustain exudation despite VEGF suppression [32]. Evidence specific to IVBr in pachychoroid is limited; e.g., Carosielli et al. [33] reported mixed responses among aflibercept-resistant eyes, implying phenotype-dependent durability. Larger studies are needed to clarify IVBr efficacy in PCV with pachychoroid.

In our cohort, greater early CCT reduction (A0 → A1) was associated with non-extension, while pachychoroid showed a similar trend but with wide CIs due to the small sample; larger studies are needed to clarify IVBr efficacy in PCV with pachychoroid.

Recent work further links pachychoroid biomarkers (CCT/CVI, pachyvessels, CVH) and early flow regression to treatment need and choroidal remodeling under anti-VEGF, including brolucizumab [34–36]. A meta-analysis also suggests faricimab is effective in PCV and may enable interval extension in a subset [37], while current guidance places anti-VEGF monotherapy as first-line with individualized adjunct PDT [38]. Although those data emphasize retinal/anatomical endpoints (e.g., CRT and polyp closure), our findings add a choroid-centric signal—early CCT thinning—as a potential predictor of dosing durability across agents.

Taken together with these reports, our data suggest that greater early CCT thinning (A0 → A1) is associated with lower odds of extension at 24 months—that is, eyes with marked early choroidal thinning were less likely to maintain >8-week intervals. Pachychoroid showed a similar direction but with wide confidence intervals. In short, CCT change (state) was more predictive than a pachychoroid label (trait) for 2-year durability after the switch. In T&E practice, a large early CCT reduction after switching may flag eyes unlikely to achieve >8-week maintenance, prompting closer surveillance and counseling about injection needs, whereas the absence of marked early thinning was more compatible with extension and a lower second-year injection burden. The few very large CCT decreases (>100 µm) clustered in heavily pre-treated eyes and likely reflect cumulative choroidal decongestion/remodeling rather than measurement error. These observations align with emerging work on pachychoroid biomarkers and choroidal remodeling under anti-VEGF therapy [28,36] and were supported by acceptable model performance (apparent AUC ≈ 0.97) and sensitivity analyses.

Looking ahead, although the faricimab meta-analysis focuses on CRT-based outcomes, our CCT-based signal suggests that early choroidal dynamics may help anticipate durability. Prospective studies should test whether CCT change similarly informs interval planning across agents—including faricimab—while integrating OCTA/CVI metrics and standardized T&E algorithms [34,37].

This study has limitations. First, one eye developed intraocular inflammation after the first brolucizumab injection and was excluded from the efficacy analyses; although retained for safety exposure counts, this may bias extension estimates and understate event rates. Second, the cohort was small (17 eyes from 16 patients) with only six extension events, yielding an events-per-variable of ~3 for our two-predictor model. To limit overfitting we prespecified the model and used Firth-penalized logistic regression with profile-likelihood 95% CIs; nonetheless, estimates—especially for pachychoroid—are imprecise. All hypothesis tests were two-sided with α = 0.05; no multiplicity adjustment was applied. We did not perform an a priori power calculation, and with six events the statistical power is limited (β likely substantial) for detecting moderate effects (e.g., ≥ 0.2–0.3 logMAR) or for precise estimation of covariate effects. A conventional design targeting 80% power (β = 0.20) for a small multivariable model would typically require ≥ ~30 events and a larger overall sample. Finally, model discrimination (apparent AUC ≈ 0.97) may be optimistic in a small sample. These findings should be regarded as hypothesis-generating; larger, prospective multicenter studies using standardized T&E protocols are warranted.

## Conclusion

In aflibercept-resistant PCV, eyes without a ≥ 40% early reduction in central choroidal thickness from baseline to the switch visit (A0 → A1) were more likely to maintain >8-week intervals at 24 months after switching to brolucizumab. Pachychoroid showed a similar tendency toward lower probability of extension, although estimates were imprecise. Early on-treatment CCT dynamics may help anticipate dosing durability under a T&E regimen and guide individualized follow-up. Prospective multicenter studies are warranted to validate these findings.

## Supporting information

S1 Data Renamed ca8fe. (XLSX)

## References

[1] Wong TY, Chakravarthy U, Klein R, Mitchell P, Zlateva G, Buggage R, et al. The natural history and prognosis of neovascular age-related macular degeneration: a systematic review of the literature and meta-analysis. Ophthalmology. 2008;115(1):116–26. doi: 10.1016/j.ophtha.2007.03.008 17675159

[2] Lim LS, Mitchell P, Seddon JM, Holz FG, Wong TY. Age-related macular degeneration. Lancet. 2012;379(9827):1728–38. doi: 10.1016/S0140-6736(12)60282-7 22559899

[3] Chakravarthy U, Pillai N, Syntosi A, Barclay L, Best C, Sagkriotis A. Association between visual acuity, lesion activity markers and retreatment decisions in neovascular age-related macular degeneration. Eye (Lond). 2020;34(12):2249–56. doi: 10.1038/s41433-020-0799-y 32066898 PMC7784949

[4] Wykoff CC, Clark WL, Nielsen JS, Brill JV, Greene LS, Heggen CL. Optimizing Anti-VEGF Treatment Outcomes for Patients with Neovascular Age-Related Macular Degeneration. J Manag Care Spec Pharm. 2018;24(2-a Suppl):S3–15. doi: 10.18553/jmcp.2018.24.2-a.s3 29383980 PMC10408401

[5] Eghøj MS, Sørensen TL. Tachyphylaxis during treatment of exudative age-related macular degeneration with ranibizumab. Br J Ophthalmol. 2012;96(1):21–3. doi: 10.1136/bjo.2011.203893 21733918

[6] Yang S, Zhao J, Sun X. Resistance to anti-VEGF therapy in neovascular age-related macular degeneration: a comprehensive review. Drug Des Devel Ther. 2016;10:1857–67. doi: 10.2147/DDDT.S97653 27330279 PMC4898027

[7] Miura M, Iwasaki T, Goto H. Intravitreal aflibercept for polypoidal choroidal vasculopathy after developing ranibizumab tachyphylaxis. Clin Ophthalmol. 2013;7:1591–5. doi: 10.2147/OPTH.S50634 23966764 PMC3743523

[8] Azuma K, Obata R, Nomura Y, Tan X, Takahashi H, Yanagi Y. Angiographic findings of ranibizumab-resistant polypoidal choroidal vasculopathy after switching to a treat-and-extend regimen with intravitreal aflibercept. Retina. 2016;36(11):2158–65. doi: 10.1097/iae.0000000000001047 27258669

[9] Hara C, Wakabayashi T, Fukushima Y, Sayanagi K, Kawasaki R, Sato S, et al. Tachyphylaxis during treatment of exudative age-related macular degeneration with aflibercept. Graefes Arch Clin Exp Ophthalmol. 2019;257(11):2559–69. doi: 10.1007/s00417-019-04456-2 31482277

[10] Broadhead GK, Keenan TDL, Chew EY, Wiley HE, Cukras CA. Comparison of agents using higher dose anti-VEGF therapy for treatment-resistant neovascular age-related macular degeneration. Graefes Arch Clin Exp Ophthalmol. 2022;260(7):2239–47. doi: 10.1007/s00417-021-05547-9 35092447

[11] Kataoka K, Itagaki K, Hashiya N, Wakugawa S, Tanaka K, Nakayama M, et al. Six-month outcomes of switching from aflibercept to faricimab in refractory cases of neovascular age-related macular degeneration. Graefes Arch Clin Exp Ophthalmol. 2023;262(1):43–51. doi: 10.1007/s00417-023-06222-x 37668741

[12] Veritti D, Lanzetta P. Triple therapy for anti-vascular endothelial growth factor nonresponders in neovascular age-related macular degeneration: impact of different photodynamic therapy parameters. Ophthalmologica. 2013;230(3):131–7. doi: 10.1159/000351651 23948986

[13] Kim JH, Kim JW, Kim CG. Comparison of 24-month treatment outcomes between as-needed treatment and switching to treat-and-extend in type 3 macular neovascularization. Sci Rep. 2022;12(1):22546. doi: 10.1038/s41598-022-25860-5 36581675 PMC9800385

[14] Cheung CMG, Lai TYY, Teo K, Ruamviboonsuk P, Chen S-J, Kim JE, et al. Polypoidal Choroidal Vasculopathy: Consensus Nomenclature and Non-Indocyanine Green Angiograph Diagnostic Criteria from the Asia-Pacific Ocular Imaging Society PCV Workgroup. Ophthalmology. 2021;128(3):443–52. doi: 10.1016/j.ophtha.2020.08.006 32795496

[15] Gal-Or O, Dansingani KK, Sebrow D, Dolz-Marco R, Freund KB. Inner choroidal flow signal attenuation in pachychoroid disease: optical Coherence Tomography Angiography. Retina. 2018;38(10):1984–92. doi: 10.1097/IAE.0000000000002051 29384997

[16] Kim H, Lee SC, Kwon KY, Lee JH, Koh HJ, Byeon SH, et al. Subfoveal choroidal thickness as a predictor of treatment response to anti-vascular endothelial growth factor therapy for polypoidal choroidal vasculopathy. Graefes Arch Clin Exp Ophthalmol. 2016;254(8):1497–503. doi: 10.1007/s00417-015-3221-x 26626772

[17] Dugel PU, Singh RP, Koh A, Ogura Y, Weissgerber G, Gedif K, et al. HAWK and HARRIER. Ophthalmology. 2021;128(1):89–99. doi: 10.1016/j.ophtha.2020.06.028 32574761

[18] Dugel PU, Koh A, Ogura Y, Jaffe GJ, Schmidt-Erfurth U, Brown DM, et al. HAWK and HARRIER: Phase 3, Multicenter, Randomized, Double-Masked Trials of Brolucizumab for Neovascular Age-Related Macular Degeneration. Ophthalmology. 2020;127(1):72–84. doi: 10.1016/j.ophtha.2019.04.017 30986442

[19] Holz FG, Dugel PU, Weissgerber G, Hamilton R, Silva R, Bandello F, et al. Single-Chain Antibody Fragment VEGF Inhibitor RTH258 for Neovascular Age-Related Macular Degeneration. Ophthalmology. 2016;123(5):1080–9. doi: 10.1016/j.ophtha.2015.12.030 26906165

[20] Venkatesh P, Takkar B, Temkar S. Clinical manifestations of pachychoroid may be secondary to pachysclera and increased scleral rigidity. Med Hypotheses. 2018;113:72–3. doi: 10.1016/j.mehy.2018.02.024 29523299

[21] Azuma K, Tanaka N, Aoki S, Kitamoto K, Ueda K, Inoue T, et al. Long-term visual outcomes in pachychoroid spectrum diseases and its associating factors of eyes with chronic central serous chorioretinopathy. Sci Rep. 2023;13(1):21876. doi: 10.1038/s41598-023-49153-7 38072873 PMC10710997

[22] Castro-Navarro V, Behar-Cohen F, Chang W, Joussen AM, Lai TYY, Navarro R, et al. Pachychoroid: current concepts on clinical features and pathogenesis. Graefes Arch Clin Exp Ophthalmol. 2021;259(6):1385–400. doi: 10.1007/s00417-020-04940-0 33057904 PMC8166704

[23] Heier JS, Brown DM, Chong V, Korobelnik J-F, Kaiser PK, Nguyen QD, et al. Intravitreal aflibercept (VEGF trap-eye) in wet age-related macular degeneration. Ophthalmology. 2012;119(12):2537–48. doi: 10.1016/j.ophtha.2012.09.006 23084240

[24] Tadayoni R, Sararols L, Weissgerber G, Verma R, Clemens A, Holz FG. Brolucizumab: A Newly Developed Anti-VEGF Molecule for the Treatment of Neovascular Age-Related Macular Degeneration. Ophthalmologica. 2021;244(2):93–101. doi: 10.1159/000513048 33197916

[25] Ueda-Consolvo T, Tanigichi A, Numata A, Oiwake T, Nakamura T, Ishida M, et al. Switching to brolucizumab from aflibercept in age-related macular degeneration with type 1 macular neovascularization and polypoidal choroidal vasculopathy: an 18-month follow-up study. Graefes Arch Clin Exp Ophthalmol. 2023;261(2):345–52. doi: 10.1007/s00417-022-05793-5 35947181

[26] Hirayama K, Yamamoto M, Honda S, Kyo A, Misawa N, Kohno T. Switching to Intravitreal Brolucizumab after Ranibizumab or Aflibercept Using Treat and Extend Regimen for Neovascular Age-Related Macular Degeneration in Japanese Patients: 1-Year Results and Factors Associated with Treatment Responsiveness. JCM. 2024;13(15):4375. doi: 10.3390/jcm13154375 39124642 PMC11312903

[27] Ota H, Takeuchi J, Nakano Y, Horiguchi E, Taki Y, Ito Y, et al. Switching from aflibercept to brolucizumab for the treatment of refractory neovascular age-related macular degeneration. Jpn J Ophthalmol. 2022;66(3):278–84. doi: 10.1007/s10384-022-00908-1 35233693

[28] Hoven E, Michelet J-T, Vettore MV, Lagali N. Choroidal thickness after anti-vascular endothelial growth factor in typical neovascular age-related macular degeneration – A systematic review and meta-analysis. Survey of Ophthalmology. 2025;70(1):86–95. doi: 10.1016/j.survophthal.2024.09.011 39374696

[29] Hikichi T, Agarie M. Reduced Vessel Density of the Choriocapillaris during Anti-Vascular Endothelial Growth Factor Therapy for Neovascular Age-Related Macular Degeneration. Invest Ophthalmol Vis Sci. 2019;60(4):1088–95. doi: 10.1167/iovs.18-24522 30901385

[30] Hood JD, Meininger CJ, Ziche M, Granger HJ. VEGF upregulates ecNOS message, protein, and NO production in human endothelial cells. American Journal of Physiology-Heart and Circulatory Physiology. 1998;274(3):H1054–8. doi: 10.1152/ajpheart.1998.274.3.h1054 9530221

[31] Sadda SR, Abdelfattah NS, Lei J, Shi Y, Marion KM, Morgenthien E, et al. Spectral-Domain OCT Analysis of Risk Factors for Macular Atrophy Development in the HARBOR Study for Neovascular Age-Related Macular Degeneration. Ophthalmology. 2020;127(10):1360–70. doi: 10.1016/j.ophtha.2020.03.031 32402555

[32] Maruyama-Inoue M, Yanagi Y, Inoue T, Kitajima Y, Kadonosono K. Importance of fluorescein angiography as a predictor of treatment response in patients with pachychoroid neovasculopathy. Sci Rep. 2024;14(1):29157. doi: 10.1038/s41598-024-79179-4 39587135 PMC11589134

[33] Carosielli M, Carnevali A, Fallico M, Pirozzi E, Chiosi F, Chronopoulos A, et al. Intravitreal Brolucizumab for Pachychoroid Neovasculopathy Associated With Chronic Central Serous Chorioretinopathy. Transl Vis Sci Technol. 2023;12(12):17. doi: 10.1167/tvst.12.12.17 38112497 PMC10732086

[34] Hoshino J, Matsumoto H, Nakamura K, Akiyama H. Predicting treatment outcomes of intravitreal brolucizumab for polypoidal choroidal vasculopathy through noninvasive assessment of polypoidal lesion blood flow with optical coherence tomography angiography. Sci Rep. 2024;14(1):961. doi: 10.1038/s41598-024-51628-0 38200216 PMC10781761

[35] Jeong A, de Jesus FR, Baek SC, Sagong M. Comparison of one-year outcomes between aflibercept and brolucizumab for treatment-naïve pachychoroid neovasculopathy. Sci Rep. 2025;15(1):13674. doi: 10.1038/s41598-025-98402-4 40259096 PMC12012145

[36] Cheung CMG, Dansingani KK, Koizumi H, Lai TYY, Sivaprasad S, Boon CJF, et al. Pachychoroid disease: review and update. Eye. 2024;39(5):819–34. doi: 10.1038/s41433-024-03253-4 39095470 PMC11933466

[37] Arnold-Vangsted A, Schou MG, Balaratnasingam C, Cehofski LJ, Chhablani J, van Dijk EHC, et al. Efficacy of intravitreal faricimab therapy for polypoidal choroidal vasculopathy: A systematic review and meta-analysis. Acta Ophthalmol. 2025;103(3):247–56. doi: 10.1111/aos.16797 39548881

[38] Iida T, Gomi F, Yasukawa T, Yamashiro K, Honda S, Maruko I, et al. Japanese clinical guidelines for neovascular age-related macular degeneration. Jpn J Ophthalmol. 2025;69(4):639–60. doi: 10.1007/s10384-025-01240-0 40658332 PMC12339655

